# Evaluation of a gene signature related to thrombotic manifestations in antiphospholipid syndrome

**DOI:** 10.3389/fmed.2023.1139906

**Published:** 2023-03-23

**Authors:** Bruna Cardoso Jacintho, Bruna de Moraes Mazetto Fonseca, Bidossessi Wilfried Hounkpe, Jose Diogo Oliveira, Ana Paula Rosa dos Santos, Camila de Oliveira Vaz, Erich Vinicius de Paula, Fernanda Andrade Orsi

**Affiliations:** ^1^School of Medical Sciences, University of Campinas (UNICAMP), Campinas, Brazil; ^2^Hematology and Hemotherapy Center, University of Campinas (UNICAMP), Campinas, Brazil; ^3^Department of Pathology, School of Medical Sciences, University of Campinas (UNICAMP), Campinas, Brazil

**Keywords:** antiphospholipid syndrome, thrombosis, gene expression, antiphospholipid antibody, mRNA

## Abstract

Thrombotic primary antiphospholipid syndrome (t-PAPS) is an acquired condition characterized by heterogeneous thrombotic manifestations, which is intriguing since venous and arterial thrombosis appear to have distinct pathogenesis. Gene expression analysis may constitute a new approach to evaluate potential similarities or differences between the clinical manifestations of t-PAPS. Recently, dysregulation of the *ANXA3, TNFAIP6, TXK, BACH2*, and *SERPINB2* genes has been associated with both arterial and venous thrombosis in the general population. Therefore, the aim of this study was to examine whether *ANXA3, TNFAIP6, TXK, BACH2*, and *SERPINB2* expression was associated with t-PAPS. Gene expression was quantified by qPCR of total leukocyte mRNA. In this case-control study, 102 t-PAPS patients, 17 asymptomatic antiphospholipid (aPL) carriers and 100 controls were evaluated. Increased expression of *ANXA3* (*P* = 0.008) and *TNFAIP6* (*P* = 0.001) and decreased expression of the *TXK* gene (*P* = 0.0001) were associated with an increased risk of t-PAPS compared to the control. *ANXA3* upregulation was more evident in cases of arterial thrombosis and multiple thrombotic events. There was no difference in the expression of these genes between triple and non-triple aPL positivity. *ANXA3*, *TNFAIP6*, *TXK, BACH2*, and *SERPINB2* expression levels were also similar between aPL carriers and controls (*P* = 0.77; *P* = 0.48; *P* = 0.08; *P* = 0.73, and *P* = 0.13, respectively). In conclusion, our results showed that genes related to hemostasis (*ANXA3*) and immunity (*TNFAIP6*, *TXK*) are dysregulated in t-PAPS compared to controls. Gene dysregulation was not detected in aPL carriers and was not related to the aPL profile, suggesting that this gene signature is related to thrombotic manifestations rather than to aPL burden. Our results suggest that innate immunity and hemostasis pathways are associated with t-PAPS at a molecular level and may play a role in disease severity.

## Introduction

Antiphospholipid syndrome (APS) is an acquired prothrombotic condition characterized by thrombosis or pregnancy complications (1) due to the presence of antiphospholipid antibodies (aPLs). These antibodies are directed against phospholipid-binding proteins, particularly beta2-glycoprotein I, found in cell membranes, including monocytes, platelets, and endothelial cells (2, 3). In primary APS (PAPS), there is no underlying systemic autoimmune disease.

The activation of platelets, monocytes, and endothelial cell membranes by aPLs leads to a hypercoagulable state (4), which is further enhanced by a secondary stimulus (second trigger), such as oral contraceptive use, infectious or inflammatory diseases, hypertension, and dyslipidemia (4, 5), resulting in thrombotic events. PAPS-associated thrombosis (t-PAPS) is one of the few thrombotic disorders that occurs indistinctly in veins, arteries or capillaries, affecting different organs and tissues (6).

Although t-PAPS is described as a single disease, arterial and venous complications differ in pathology, clinical course, treatment and prognosis, suggesting that different mechanisms underlie these thrombotic complications. Recently, studies aimed at evaluating the vascular impairment of t-PAPS have shown that aPLs can induce changes in the expression of genes associated with procoagulant and proinflammatory markers, leading to a prothrombotic state (7). Therefore, gene expression analysis may be a new approach to study the etiology of thrombosis in APS.

Recently, the profile of genes associated with venous and arterial thrombosis in the general population (not associated with APS) was evaluated by meta-analysis (8). In this study, five databases were analyzed and gene expression levels in whole blood from patients with venous thrombosis or arterial cardiovascular disease were compared with gene expression levels in controls. A total of 124 genes showed differential expression levels between these diseases. A further 473 genes had altered expression in the same direction in venous and arterial disease (168 upregulated and 305 downregulated). Of these, six genes were most strongly associated with both venous and arterial thrombosis: G0S2, BCL2A1, TNFAIP6 (upregulated), CLIC3, BACH2, and TXK (downregulated). The results of the meta-analysis suggest that a specific gene profile may be associated with the occurrence of both arterial and venous thrombosis in patients without APS.

Comparing the results of the meta-analysis described above (8) with the results of previous APS studies (9, 10), we found that the genes ANXA3, TNFAIP6, TXK, BACH2, and SERPINB2 are differentially expressed in both venous and arterial thrombosis as well as in APS compared to healthy subjects. Considering that the heterogeneity of t-PAPS manifestations cannot be explained by a single prothrombotic mechanism, it is possible that a gene signature underlies the occurrence and severity of thrombosis in PAPS.

In this context, the primary aim of this study was to determine the expression in patients with t-PAPS of genes previously associated with both arterial and venous thrombosis in the general population. We also investigated whether these genes are dysregulated in asymptomatic aPL carriers.

## Materials and methods

### Study design and participant selection

In this case-control study, we initially included consecutive patients with t-PAPS treated at the Hematology and Hemotherapy Center of the University of Campinas–Hemocentro-UNICAMP and individuals without a history of thrombosis (controls). Subsequently, asymptomatic aPL carriers were also included in the study. The study was approved by the local Ethics Committee (CAAE: 14902019.1.0000.5404), and the procedures were only performed after a signed informed consent form was obtained from patients.

We included patients with confirmed APS, a history of at least one previous thrombotic event within the previous 10 years. Cancer, pregnancy, isolated obstetric APS, systemic autoimmune diseases and infectious diseases were reasons for exclusion. APS diagnosis was confirmed based on the Sydney criteria (11).

Individuals with no previous history of thrombotic events and negative aPL results (called controls) were selected. Controls were recruited among people from the same geographic region as the patients and matched to them according to sex and age group (± 5 years). Controls were selected among students, university staff and voluntary blood donors. Previous venous thrombosis, stroke, myocardial infarction, neoplasia, and pregnancy were reasons for exclusion. Individuals with persistently positive aPL results and no previous history of thrombosis or obstetric complications suggestive of APS were selected as asymptomatic aPL carriers. aPL carriers group was composed of asymptomatic patients under investigation of prolonged activated partial thromboplastin time (aPTT), immune thrombocytopenia (ITP) patients treated at the same center as t-PAPS or among healthy controls whose aPL tests were positive. Only individuals without signs of infection or acute inflammation were tested for aPL. As part of the diagnosis criteria (11), aPL positivity was confirmed after a 12-week interval. Exclusion criteria were the same as for healthy controls.

Patients, controls and aPL carriers answered a questionnaire about their demographic data, habits, health status and use of medication. Information regarding clinical data was also obtained by consulting the electronic medical record.

### Laboratory procedures and mRNA expression of selected genes

Blood samples were collected upon inclusion into anticoagulant ethylenediaminetetraacetic acid (EDTA)-containing tubes. The samples were processed within 2 h of collection, the red blood cells were lysed with a buffer containing ammonium chloride and ammonium bicarbonate and centrifuged in a refrigerated centrifuge at 4°C, and the leukocyte layer was separated for RNA isolation. Samples were stored at −80°C until analysis.

As mentioned above, the relative expression of the five genes of interest was investigated in t-PAPS patients. These genes were selected among those described in a recent meta-analysis that evaluated genes with concordant regulation in arterial and venous thrombosis (8). The ones most associated with arterial and venous thrombosis, three upregulated (ANXA3, TNFAIP6, and SERPINB2) and two downregulated (TXK and BACH2), were selected to be validated in thrombotic APS.

TRIzol™ reagent (Invitrogen™) was used to isolate mRNA from total leukocytes, following the manufacturer’s instructions. Next, samples were transcribed into cDNA using the RevertAid First Strand cDNA Synthesis Kit^®^ (Thermo Scientific™). The order and steps of incubation were performed according to manufacturer’s instructions.

The primers used in the real-time quantitative PCR (qPCR) reaction were designed in the Gene Runner program (Supplementary Table 1). We verified the specificity, dissociation temperature, and formation of secondary structures of the primer pair. The slope of a standard curve is commonly used to estimate the amplification efficiency of a real-time qPCR. The estimated efficiency (EFF) calculation for a real-time PCR assay is EFF = (10−1/*slope*−1)×100 (12). An *ANXA3* calibration curve was obtained at a primer concentration of 150 nM [slope −3.4, correlation coefficient (CC) = 0.99 and EFF 93%], *TNFAIP6* at 150 nM [slope −3.3, CC = 0.99, and EFF 99%], *TXK* at 150 nM [slope −3.3, CC = 0.99 and EFF 98%], *BACH2* at 150 nM [slope −3.4, CC = 0.99 and EFF 95%], *SERPINB2* at 300 nM [slope −3.3, CC = 0.98 and EFF 99%], *RHOA* at 150 nM [slope −3.2, CC = 0.99 and EFF 104%], and *EEF2* at 150 nM [slope −3.3, CC = 0.99 and EFF 99%]. Relative expression of genes was normalized using *EEF2* and *RHOA* as reference genes. Real-time amplification detection was performed using a real-time PCR thermocycler (QuantStudio 6 and 7–Thermo Fisher Scientific). Negative controls (NTC–No Template Control) were pipetted into all plates (wells containing only SYBR and primers) without the addition of cDNA to verify the absence of contamination. Reactions were performed in 96-well plates (Applied Biosystems) sealed with optical adhesives (Applied Biosystems). The relative expression was denoted as an “arbitrary unit” (AU).

### Statistical analysis

Descriptive analyses were performed using frequency tables for categorical variables and position and dispersion measures for numerical variables, which were expressed as the mean and standard deviation (if the variable was normally distributed) or median and interquartile range [IQR] (if the variable was non-normally distributed).

To compare relative mRNA expression between two groups, we used either a parametric *t*-test or a non-parametric Mann-Whitney test, depending on the distribution of values (normal vs. non-normal). For the correlation between two variables, a non-parametric test of the Spearman rank correlation coefficient was used.

Logistic regression models were used to evaluate the association between relative *ANXA3*, *TNFAIP6*, *TXK*, *BACH2*, and *SERPINB2* mRNA expression and t-PAPS. Next, patients with t-PAPS were divided into 3 subgroups: (i) venous thrombosis or arterial thrombosis; (ii) single thrombotic event or multiple thrombotic events; and (iii) non-triple aPL-positive or triple aPL-positive. Logistic regression models were used to identify the association between mRNA expression and the subgroups, using the control group as a reference. The results were expressed as odds ratios (ORs) and 95% confidence intervals (CIs).

SPSS version 25.0 for Windows (SPSS Inc., Chicago, IL, USA) was used for statistical analyses. GraphPad Prism version 8.0 (GraphPad Software Inc., La Jolla, CA, USA) was used for graph plotting.

## Results

### Patient’s selection and clinical characteristics

**FIGURE 1:**
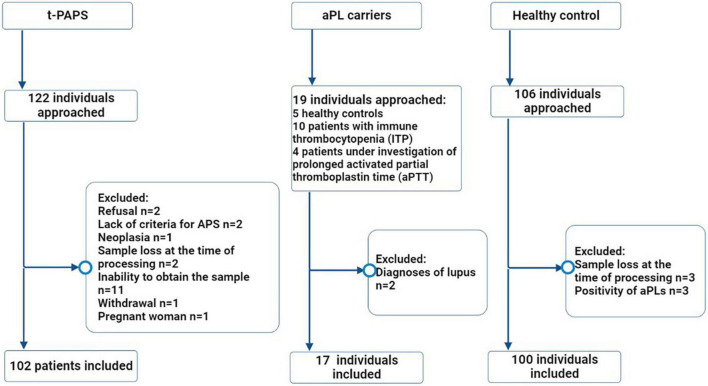
Flowchart of the selection process of study participants. Legend: The flowchart illustrates the number of individuals who were approached (*n* = 247), the excluded participants and the reasons for exclusions, the final number of individuals who were included in the study (*n* = 219) and the distribution between the groups. t-PAPS, primary thrombotic APS; aPL, antiphospholipid antibodies; *n, absolute number.

A total of 219 participants were included in the study: 102 t-PAPS, 100 healthy controls and 17 asymptomatic aPL carriers. Figure 1 illustrates the flowchart of the selection process of controls and patients, as well as the reasons for exclusion from the study.

Participants’ demographic, clinical and laboratory features are shown in Table 1. Cardiovascular risk factors were more prevalent in t-PAPS than in asymptomatic aPL carriers or controls. Most t-PAPS and asymptomatic aPL carriers were positive for lupus anticoagulant, while 22% of t-PAPS and 29% of asymptomatic aPL carriers were triple positive for aPLs. Details on APS-associated thrombotic events are presented in Table 2. The median age at the time of the first thrombosis was 35 years old [IQR 25–48], 60% of thrombotic events were unprovoked, 72% were venous and 28% arterial thrombosis. The first thrombotic event was deep vein thrombosis in 42% of the patients, ischemic stroke in 25%, pulmonary thromboembolism (PE) in 15%, and cerebral venous thrombosis in 12%. Portal vein thrombosis, acute myocardial infarction, intestinal thrombosis and retinal thrombosis occurred in 6% of the patients. A total of 36% of the patients had multiple thrombotic events. The median time elapsed between thrombotic events was 47 months [IQR 12.0–91.5].

**TABLE 1:** Demographic, clinical and laboratory features of the study participants.

Parameters	t-PAPS (*n* = 102)	aPL carriers (*n* = 17)	Controls (*n* = 100)
Age, median (IQR)	40 (31–53)	36 (24–59)	39 (29–49)
Woman, *n* (%)	65 (64)	14 (82)	67 (67)
Tabagism, *n* (%)	19 (19)	1 (6)	1 (1)
Hypertension, *n* (%)	33 (32)	2 (12)	6 (6)
Dyslipidemia, *n* (%)	34 (33)	1 (6)	11 (11)
Diabetes, *n* (%)	7 (7)	2 (12)	6 (6)
Obesity (BMI ≥ 30.0 kg/m^2^), *n* (%)	39 (38)	1 (6)	9 (9)
Continuous medication use, *n* (%)	95 (93)	10 (59)	33 (33)
Statin use, *n* (%)	28 (27)	1 (6)	7 (7)
ASA use, *n* (%)	26 (25)	1 (6)	0 (0)
Use of anticoagulants*, *n* (%)	80 (78)	0	0
Family history**, *n* (%)	59 (58)	11 (65)	40 (40)
Gestational complication***, n (%)	17 (26)	0	0 (0)
Abortion, *n* (%)	19 (29)	1 (7)	2 (3)
LAC, *n* (%)	84 (82)	12 (71)	0
aCL IgM, *n* (%)	15 (15)	4 (24)	0
aCL IgG, *n* (%)	44 (43)	8 (47)	0
Anti-β2GPI IgM, *n* (%)	35 (34)	8 (47)	0
Anti-β2GPI IgG, *n* (%)	39 (38)	9 (53)	0
Triple positivity for aPL, *n* (%)	22 (22)	5 (29)	0

n, absolute number; t-PAPS, primary thrombotic antiphospholipid syndrome; aPL, antiphospholipid antibodies; ASA, acetylsalicylic acid; LAC, lupus anticoagulant; aCL, anticardiolipin; Antiβ2GPI, anti-beta2-glycoprotein-1 antibody; IgM, immunoglobulin M; IgG, immunoglobulin G.

*Most patients were using warfarin, and only two patients in the primary APS group were using rivaroxaban at the time of inclusion in the study.

**Family histories were deep venous thrombosis, arterial occlusion, stroke, and acute myocardial infarction.

***Pregnancy complications were fetal loss, intrauterine growth retardation (IUGR), preeclampsia, eclampsia or HELLP syndrome. Dyslipidemia was characterized as LDL-C levels above 100 mg/dL, HDL-C levels below 40 mg/dL (men), or 50 mg/dL (women), TG levels above 200 mg/dL, non-HDL-C levels above 130 mg/dL and TC levels above 190 mg/dL (31).

**TABLE 2:** Thrombotic manifestation in patients with t-PAPS.

	t-PAPS (*n* = 102)
Number of thrombotic events, median (IQR)	1 (1–2)
Age at thrombotic event, median (IQR)	35 (25–48)
**Characterization of the thrombotic event**	
Provoked, *n* (%)	41 (40)
Unprovoked, *n* (%)	61 (60)
**Type of thrombosis**	
Arterial, *n* (%)	29 (28)
Venous, *n* (%)	73 (72)
**Thrombosis site (first event)**	
Deep vein thrombosis, *n* (%)	43 (42)
Ischemic stroke, *n* (%)	25 (25)
PE, *n* (%)	15 (15)
cerebral venous thrombosis, *n* (%)	12 (12)
Other sites*, *n* (%)	7 (6)
Multiple thromboses, *n* (%)	37 (36)
Time elapsed between thrombotic events in months, median (IQR)	47 (12.0–91.5)
**Site of recurrence**	
Deep vein thrombosis, *n* (%)	15 (14)
Ischemic stroke, *n* (%)	8 (8)
PE, *n* (%)	7 (7)
Other sites*, *n* (%)	7 (7)

n, absolute number; IQR, interquartile range; PE, pulmonary embolism.

*Other sites were portal vein thrombosis, acute myocardial infarction, intestinal thrombosis and retinal thrombosis.

### *ANXA3*, *TNFAIP6*, and *TXK* are differentially expressed in patients with t-PAPS as compared to the control group

**FIGURE 2:**
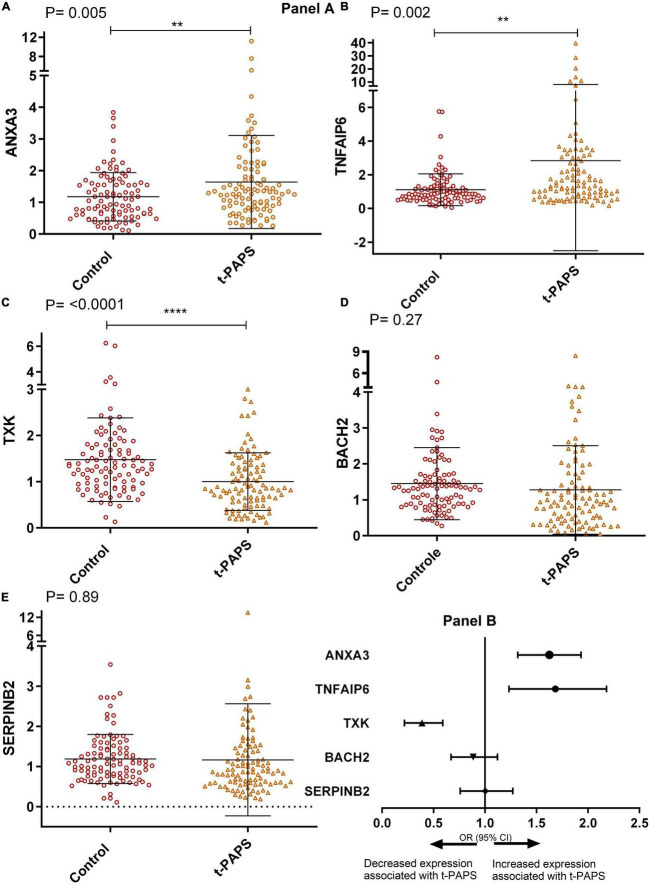
Panel **(A)** comparative graphs of the expression of genes related to thrombosis. Relative expression in total leukocytes of the genes: **(A)** *ANXA3*, **(B)** *TNFAIP6*, **(C)** *TXK*, **(D)** *BACH2*, and **(E)** *SERPINB2*. To illustrate the graphs above, mean and SD were used, and *P*-values were calculated using Student’s *t*-test. t-PAPS = primary thrombotic APS. The mean *ANXA3* mRNA expression was 1.17 AU [95% CI 1.03–1.33] in controls and 1.64 AU [95% CI 1.36–1.93] in t-PAPS [*P* = 0.005]. The mean *TNFAIP6* expression was 1.10 AU [95% CI 0.92–1.29] in controls and 2.84 [95% CI 1.89–3.89] in t-PAPS [*P* = 0.002]. The mean *TXK* expression was 1.47 AU [95% CI 1.30–1.66] in controls and 1.00 AU [95% CI 0.88–1.12] in t-PAPS [*P* = < 0.0001]. The mean *BACH2* expression was 1.45 AU [95% CI 1.25–1.65] in controls and 1.27 AU [95% CI 1.03–1.52] in t-PAPS [*P* = 0.27]. Finally, the mean *SERPINB2* expression was 1.19 AU [95% CI 1.07–1.31] in controls and 1.17 AU [95% CI 0.89–1.44] in t-PAPS [*P* = 0.89]. Panel **(B)** association of *ANXA3*, *TNFAIP6*, *TXK*, *BACH2*, and *SERPINB2* relative expression with t-PAPS diagnosis. Increased *ANXA3* mRNA expression [OR = 1.57; 95% CI 1.13–2.18; *P* = 0.008] and *TNFAIP6* expression [OR = 1.64; 95% CI 1.23–2.18; *P* = 0.001] and decreased *TXK* mRNA expression [OR = 0.36; 95% CI 0.22–0.59; *P* = 0.0001] were associated with a higher risk of t-PAPS compared with controls. *BACH2* and *SERPINB2* mRNA expression was not associated with t-PAPS [OR = 0.87; 95% CI 0.67–1.12; *P* = 0.28 and OR = 0.98; 95% CI 0.75–1.3; *P* = 0.89, respectively]. OR, odds ratio; CI, confidence interval; t-PAPS, primary thrombotic APS. **P < 0.01 and ****P = < 0.0001.

When comparing relative mRNA expressions, t-PAPS patients had higher levels of *ANXA3* and *TNFAIP6* and lower levels of *TXK* than healthy controls [*P* = 0.005; *P* = 0.002, and *P* = < 0.0001, respectively]. There were no statistically relevant differences between groups in *BACH2* and *SERPINB2* levels (Figure 2, panel A). Consequently, increased *ANXA3* and *TNFAIP6* mRNA expression and decreased *TXK* mRNA expression were associated with a higher risk of t-PAPS (Figure 2, panel B).

**FIGURE 3:**
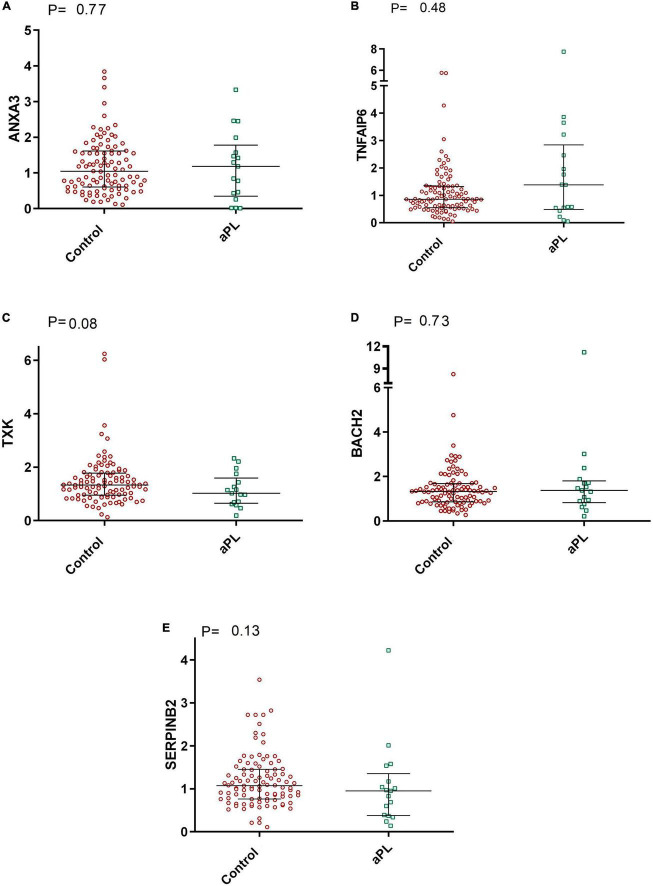
Comparative graphs of the expression of genes related to thrombosis. Legend: Relative expression of the genes **(A)** *ANXA3*, **(B)** *TNFAIP6*, **(C)** *TXK*, **(D)** *BACH2*, and **(E)** *SERPINB2* in total leukocytes was evaluated in aPL carriers compared to controls. Median and IQR were used by the Mann-Whitney test. aPL, asymptomatic aPL carriers (aPL +). The median relative expression of the *ANXA3* gene was 1.04 (IQR 0.60–1.61) in control subjects and 1.18 (IQR 0.34–1.78) in aPL + subjects. The relative expression of *TNFAIP6* was 0.86 (IQR 0.55–1.32) in controls and 1.38 (IQR 0.49–2.84) in aPL +. The relative expression of *TXK* was 1.33 (IQR 0.95–1.77) in controls and 1.02 (IQR 0.65–1.59) in aPL + patients. *BACH2* relative expression was 1.32 (IQR 0.86–1.68) in controls and 1.38 (IQR 0.82–1.80) in aPL +. Finally, the relative expression of *SERPINB2* was 1.07 (IQR 0.76–1.45) in controls and 0.95 (IQR 0.38–1.35) in aPL +.

In addition, as shown in Figure 3, the mRNA expression levels of *ANXA3*, *TNFAIP6*, *TXK*, *BACH2*, and *SERPINB2* were similar between asymptomatic aPL carriers and controls. [*P* = 0.77; *P* = 0.48; *P* = 0.08; *P* = 0.73, and *P* = 0.13, respectively].

### Elevated *ANXA3* expression tends to be associated with arterial thrombosis and multiple thrombotic events

**FIGURE 4:**
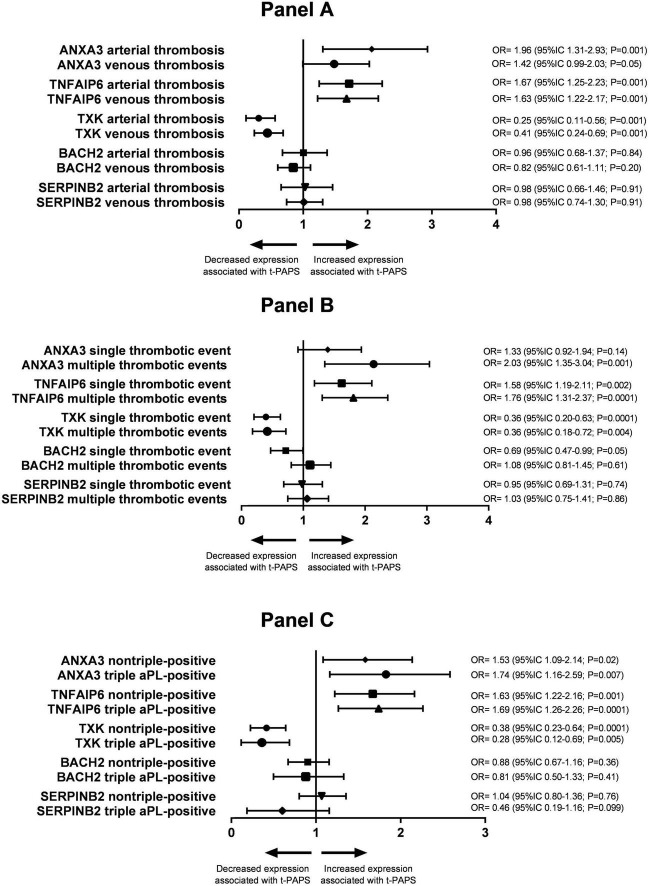
Panel **(A)** association between the relative expression of the study genes and arterial or venous thrombosis in t-PAPS. Panel **(B)** association between the relative expression of the study genes and a single thrombotic event or multiple events in t-PAPS. Panel **(C)** association between the relative expression of the study genes and non-triple-positive or triple aPL-positive subgroups. OR, odds ratio; CI, confidence interval; t-PAPS, primary thrombotic APS.

The association of *ANXA3*, *TNFAIP6*, *TXK*, *BACH2*, and *SERPINB2* mRNA expression with the type of thrombosis (arterial or venous) in t-PAPS is shown in Figure 4, Panel A. *ANXA3* mRNA upregulation was more evident in arterial thrombosis than in venous thrombosis when compared with controls. The relative expression of the other genes did not differ significantly between arterial and venous t-PAPS compared to controls.

Next, the association of *ANXA3*, *TNFAIP6*, *TXK*, *BACH2*, and *SERPINB2* mRNA expression with the number of previous thrombosis events (single or multiple thrombosis) is shown in Figure 4, Panel B. *ANXA3* mRNA upregulation was, again, more pronounced in multiple thrombosis than in single thrombosis when compared to controls. *BACH2* mRNA downregulation was more pronounced in single thrombosis than in multiple thrombosis compared to controls. The relative expression of the remaining genes did not differ between single and multiple thrombotic events compared to controls.

Finally, *ANXA3*, *TNFAIP6*, *TXK*, *BACH2*, and *SERPINB2* mRNA expression levels were similar between non-triple-positive and triple aPL-positive t-PAPS (Figure 4, Panel C).

## Discussion

Antiphospholipid syndrome is a good clinical model to evaluate similarities and differences between venous and arterial thrombosis, as the disease is characterized by thrombotic manifestations in multiple vascular sites. This heterogeneous presentation is intriguing because venous and arterial thrombosis appear to have different pathogenesis. While stasis-related mechanisms, such as immobilization and congestive heart failure, and hypercoagulability-related mechanisms, such as familial thrombophilia and oral contraceptive use, play a fundamental role in the occurrence of venous thrombosis (13), arterial thrombosis occurs in high-flow sites and is mainly dependent on vascular integrity and platelet function (14, 15). However, some clinical aspects may be common to both arterial and venous thrombosis. Age, obesity and hypertension are known risk factors for atheromatous disease and arterial thrombosis. These factors may also contribute to the development of venous thrombosis (16, 17).

Much remains to be understood about common pathologic mechanisms leading to heterogeneous thrombotic manifestations in APS. One way to investigate common or diverse causes of the heterogeneous clinical presentation of APS is to study the genetic profile of the disease. Translational studies have allowed the identification of gene sets (or signatures) associated with PAPS (18) and secondary APS (SAPS) (19), as well as the expression profile of genes that differentiate thrombotic APS from APS with obstetric complications (9).

For example, obstetric APS is characterized by differentially expressed genes involved in cell adhesion, extracellular matrix, embryogenesis, and skeletal development, whereas thrombotic APS is characterized by differentially expressed genes involved in pro-inflammatory cytokine production, cellular response to stress, oxidative stress, and cellular homeostasis (9). PAPS and SAPS share the expression of genes involved in the type 1 interferon signature, inflammation and atherosclerosis (10), whereas PAPS differs from systemic lupus erythematosus (SLE) by the expression of genes related to mitochondrial function, oxidative stress and antioxidant capacity (10, 18, 19). We recently observed that genes related to the type 1 interferon signature were overexpressed in t-SAPS, but not in t-PAPS, compared to controls (20), suggesting that the inflammatory profile is different between these two presentations of APS. A recent meta-analysis identified 16 genes differentially expressed in t-PAPS compared to controls that were expressed in 32 different tissues, which may explain the fact that thrombosis in APS occurs in any vascular site (18). Thus, measurement of gene expression has been used to develop new biological concepts, refine disease classification, improve diagnostic and prognostic accuracy, and identify new molecular targets for treatment.

We chose to quantify *ANXA3*, *TNFAIP6*, *TXK*, *BACH2*, and *SERPINB2* mRNA expression in t-PAPS based on the results of a meta-analysis using a bioinformatics panel to explore the differences and similarities between venous thromboembolism (VTE) and cardiovascular disease. The results of the meta-analysis represented the first comparison of venous and arterial thrombosis at the transcriptomic level, analyzing gene expression datasets from microarray studies involving human patients with cardiovascular disease or VTE in the Gene Expression Omnibus (GEO) public repository (8); however, they needed to be validated in a real-world cohort.

In this context, this study analyzed the expression of genes previously associated with venous and arterial thrombosis in the general population in t-PAPS and asymptomatic aPL carriers. Our results showed that the expression of *ANXA3* and *TNFAIP6* is upregulated and *TXK* is downregulated in t-PAPS compared to controls. Furthermore, increased *ANXA3* mRNA expression is more pronounced in patients with arterial thrombosis or multiple thromboses, suggesting that *ANXA3* is not only associated with thrombosis in PAPS but also with the severity of the thrombotic event.

*ANXA3*, *TNFAIP6*, *TXK*, *BACH2*, and *SERPINB2* are mainly associated with immunity and hemostasis. *ANXA3* plays a role in regulation of cell growth, regulation of endothelial cell migration, maintenance of cellular phospholipids and signal transduction pathways and has been associated with stroke in animal studies (21). *TNFAIP6* is involved in cell-cell and cell-matrix interactions in inflammation and cancer, and its increased expression in t-PAPS may be explained by the inflammatory process of the disease (8, 22). *TXK* acts on pathways related to the blood-brain barrier, transmigration of immune cells, and regulates the development, function, and differentiation of T-cells and NKT cells (23, 24). *BACH2* is responsible for immune regulation, mainly related to the adaptive immune response, and has been implicated in B-cell function (25, 26). *SERPINB2* regulates the production of plasminogen activator inhibitor-2 and is directly related to fibrinolysis pathways (8, 27). Our study showed that t-PAPS is associated with dysregulation of genes related to hemostasis (*ANXA3*) and inflammation/immunity (*TNFAIP6* and *TXK*). *ANXA3* has also been associated with the occurrence of arterial thrombosis and multiple thrombotic events. Interestingly, this gene has been shown to be upregulated in rodent models of ischemic stroke in animal studies (28). Therefore, our results suggest that innate immunity and hemostasis are associated with thrombotic manifestations of t-PAPS at the molecular level, with the potential to detect more severe forms of the disease.

In the meta-analysis that inspired this study, the association between *BACH2* and *SERPINB2* with arterial and venous thrombosis was weaker than that of the other genes. Moreover, from a biological point of view, *SERPINB2* regulates the plasminogen activator-2 inhibitor pathway, which is responsible for a fibrinolytic mechanism that is less associated with APS-related hypercoagulability. *BACH2* is a gene related to adaptive/humoral immunity, it has been described as a regulator of adaptive immunity through T-cell maintenance and B-cell maturation. In t-PAPS patients, we observed a dysregulation of genes associated with innate immunity.

The estimated incidence of thrombosis in asymptomatic aPL+ carriers is approximately 1% per year (29). Present in up to 5% of the general population, aPL antibodies may either be clinically irrelevant or confer an increased risk of thrombotic manifestations (30). In this context, we included the group of asymptomatic aPL carriers in this study to evaluate whether the molecular alterations observed in t-PAPS are also present in asymptomatic aPL carriers. Interestingly, the mRNA expression levels of *ANXA3*, *TNFAIP6*, *TXK*, *BACH2*, and *SERPINB2* were similar between asymptomatic aPL carriers and controls. Gene expression was not affected by aPL profile, particularly aPL triple positivity. Taken together, these results suggest that dysregulation of *ANXA3*, *TNFAIP6*, and *TXK*, although associated with thrombotic manifestations of PAPS, is not due to the presence or burden of aPL antibodies.

Limitations of our study include the fact that we included a group of patients treated in a tertiary care center and, therefore, it is possible that our patients presented with a more severe clinical profile. The cohort is heterogeneous because of the variable duration of the disease; however, there was no correlation between the time elapsed since diagnosis of t-PAPS and the relative expression of the genes (data not shown). Data collection was performed retrospectively by consulting electronic medical records, which may contain imprecise information.

## Conclusion

The results demonstrated that genes related to both innate immunity and hemostasis were associated with thrombotic manifestations in PAPS and with disease severity (arterial thrombosis and multiple thrombotic events), independent of the aPL profile. Therefore, our findings suggest a molecular link between hemostasis, immune response and thrombosis in PAPS, providing new insights into the mechanisms underlying the development of thrombosis in PAPS.

## Data availability statement

The original contributions presented in this study are included in the article/Supplementary material, further inquiries can be directed to the corresponding author.

## Ethics statement

The studies involving human participants were reviewed and approved by the CEP UNICAMP–Campus Campinas. The patients/participants provided their written informed consent to participate in this study.

## Author contributions

BJ performed the laboratory and statistical analyses and drafted the manuscript. BM, BH, JO, AS, CV, and EP revised the manuscript. FO designed the study and the analyses and revised the manuscript. All authors contributed to the article and approved the submitted version.
