# Supplementary material for: Evaluation of a gene signature related to thrombotic manifestations in antiphospholipid syndrome
Source: Front Med (Lausanne). 2023 Mar 23;10:1139906. doi: 10.3389/fmed.2023.1139906 (PMC10076702; doi:10.3389/fmed.2023.1139906)
Supplement: Supplementary file 1 [file Data_Sheet_1.PDF]

Supplementary Table 1 - Amplified fragment sequence of each primer pair for qPCR for genes associated with venous and arterial thrombosis and reference genes.

Genes	Primers sequence
ANXA3 – F	5′-GTTGGACACCGAGGAACAG-3’
ANXA3 – R	5′-GACCTCTCAGTCAGAATGCTG-3’
TNFAIP6 – F	5’-GTGGGAAGATACTGTGGAGATG-3’
TNFAIP6 – R	5’-CCTCCAGCTGTCACTGAAG-3’
TXK – F	5’-CATAGGGATTTGGCGGCA-3’
TXK – R	5’-CACTTGATTGGGAACTTGGCTC-3’
BACH2 – F	5′- CTGCAGCATGAACAAGGTG -3’
BACH2 – R	5′- GAGAAGGCTCTGGCAATCC -3’
SERPINB2 – F	5’-GATGGCCAAGGTGCTTCAG-3’
SERPINB2 – R	5’-GAGAGAGCGGAAGGATGAATG-3’
EEF2 – F	5’ TGAACAAGATGGACCGCG 3’
EEF2 – R	5’ GGATCGATCATGATGTTGCC3’
RHOA –F	5’TCTCCTACCCAGATACCGATG3’
RHOA – R	5’ACCGGCTCCTGCTTCATCT3’

Legend: F=Forward; R= Reverse
